# Insights into the Establishment of the Manila Clam on a Tidal Flat at the Southern End of an Introduced Range in Southern California, USA

**DOI:** 10.1371/journal.pone.0118891

**Published:** 2015-03-20

**Authors:** Drew M. Talley, Theresa Sinicrope Talley, Alexander Blanco

**Affiliations:** 1 Department of Marine Science and Environmental Studies, University of San Diego, San Diego, California, United States of America; 2 California Sea Grant Extension Program, Scripps Institution of Oceanography, University of California San Diego, La Jolla, California, United States of America; Université du Québec à Rimouski, CANADA

## Abstract

Coastal ecosystem modifications have contributed to the spread of introduced species through alterations of historic disturbance regimes and resource availability, and increased propagule pressure. Frequency of occurrence of the Manila clam (*Venerupis phillipinarum*, Veneridae) in Southern California estuaries has increased from absent or sparse to common since the mid-1990s. Potential invasion vectors include seafood sales and aquaculture, and spread from established northern populations over decades. The clam’s post-settlement habitat preferences are, however, uncertain in this region. Our project aimed to identify factors associated with established patches of the clam within a bay toward the southern end of this introduced range. During summer 2013, we sampled 10 tidal flat sites in Mission Bay, San Diego; each containing an area with and without hard structure (e.g., riprap, boulders). We measured likely environmental influences (e.g., sediment variables, distance to ocean). Manila clam densities across the bay were most strongly associated with site, where highest densities were located in the northern and/or back halves of the bay; and weakly correlated with lower porewater salinities. Within sites, Manila clam density was enhanced in the presence of hard structure in most sites. Prevailing currents and salinity regimes likely contribute to bay wide distributions, while hard structures may provide suitable microhabitats (refuge from predators and physical stress) and larval entrapment within sites. Results provide insights into decisions about future shoreline management efforts. Finally, we identify directions for future study to better understand and therefore predict patterns of establishment of the Manila clam in the southern portion of its introduced range.

## Introduction

Humans play key roles in the spread and subsequent establishment of species from native to introduced ranges around the world [1]. Estuarine systems are, in particular, highly susceptible to invasion [2]. Development and use of these coastal ecosystems change historic disturbance regimes and resource availability, and increase propagule pressure raising the likelihood of establishment and eventual spread of non-native species (e.g., [2–4]).

The Manila clam (*Venerupis phillipinarum*, A. Adams and Reeve, 1850), also known as the Japanese littleneck, was introduced from Asia in the 1930s to the northwest coast of North America along with Pacific oysters [5],[6]. Expansion throughout the northwest coast, from British Columbia to Northern California, has been due to planktonic larval dispersal, accidental introductions with transplanted oysters, and intentional introductions of transplanted clams for aquaculture [5–9]. While establishment and spread tend to be heaviest from San Francisco Bay northward [2],[10], the Manila clam appears to be increasing in Southern California based on comparisons among recent surveys, in which the clam was found to be relatively common (e.g., [11],[12]), and earlier studies conducted in the same areas that did not find the clam [2],[13],[14]. Vectors of spread in the southern end of this range probably include sale of live clams, aquaculture [15], and transport from the northern established populations. Influences on establishment within Southern California bays, however, remain uncertain.

In San Francisco Bay, where populations are relatively well studied [6],[10], and in Colorado Lagoon, Los Angeles County [12], the clam ranges from subtidal to high intertidal elevations. Intertidally, it is found on coarse sandy mud, gravel and cobble, and subtidally on oyster shell beds [10]. It survives salinities ranging from 13.5–35 PSU [16], grows and reproduces best within water salinities of 24–31 PSU, and can tolerate [6] or even prefer [16][17] salinities between 10–15 PSU. The clam, like other infaunal bivalves, may also be sensitive to porewater salinities [18],[19]. The surface sediments inhabited by the clam likely track the maxima of bottom water salinities [20],[21] so that only porewater water samples are analyzed in this study. In Mission Bay, as with many other embayments in Southern California, there is relatively little tidal flat area thereby limiting expanses of coarse substrate, and there are few to no dense patches of oysters or shell hash [12],[22],[23]. Salinities tend to range from seawater (34–35 PSU) near the mouth to hypersaline conditions toward the back of the bay (38–40 PSU), but this inverse gradient varies with season (i.e., amounts of precipitation and temperature) and proximity to freshwater inputs [21]. Despite the seeming lack of optimal habitat, the Manila clam has anecdotally been reported around the bay (e.g., [11],[24], C. Gramlich pers. comm.), in particular on soft substrate located next to hard structures, such as immediately adjacent to riprap and boulders (T.S. Talley pers. obs.).

The goal of our study was to increase our understanding of influences on the distribution of the Manila clam in the southern portion of its introduced range. To meet this goal, we tested two hypotheses. First, that Manila clam density throughout Mission Bay would increase with lower salinities and/or coarser sediments. Second, that within sites there would be higher clam densities on tidal flats with hard structure as compared with those without structure.

## Materials and Methods

**Fig 1:**
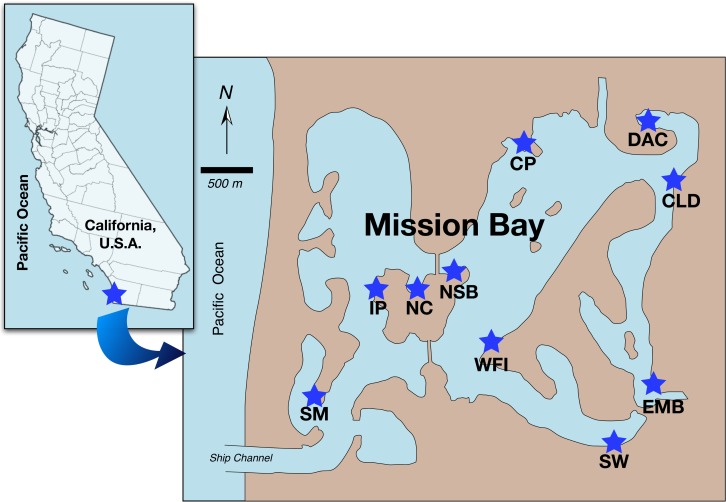
Map of intertidal benthic study sites within Mission Bay, San Diego, California, USA. SM = South Mission Bay, IP = Island Point, NC = North Cove, NSB = North Ski Beach, WFI = Fiesta Island, CP = Crown Point, DAC = D’Anza Cove, CLD = Clairemont Drive, EMB = East Mission Bay Drive, SW = Sea World. The Manila clam was found in all sites except SM and WFI.

Between June 20^th^ and July 12^th^ 2013, we sampled ten tidal flats around Mission Bay, San Diego, California, USA (32° 46’ 40.8”, 117° 13’ 30”), which has an average spring tide range of 1.7 meters. The tidal flats were broadly chosen to represent the geographic range of the bay, with specific sample locations haphazardly selected within region. Tidal flats were 2.2 to 8.7 km from the mouth of the bay as the water flows (Fig. 1). Each tidal flat consisted of 75–100 m stretches with no hard structure (≤5% rock or debris) and adjoining, similar sized stretches of tidal flat containing hard structure (≥75% cover of boulders or riprap). The riprap and boulders were mostly granite and/or manmade stone blocks ranging in size from 0.1–0.7 cubic meters and piled on the edge of the bay from about 0.75–1 m to over 2 m above mean lower low water (MLLW). The tidal flat surfaces were located at the same tidal elevation (0.75–1 m above MLLW), and the paired, adjacent flats faced the same direction.

All sites except Crown Point (CP, Fig. 1) were open to public access within Mission Bay Park so did not require special permission to enter sites. The Crown Point salt marsh creek fell within the boundaries of the University of California Kendall Frost Marsh Reserve and permission to conduct the sampling was granted from Isabelle Kay, Reserves Manager at the U.C. Natural Reserve System. All collections were conducted under the California Fish and Wildlife Scientific Collection Permit administered to T.S. Talley (Permit number SC-5295).

Within each of the 10 tidal flats, six sampling points were established: three points on tidal flats with hard structure present and three on stretches with no structure. Sampling points were 25–35 m apart and points in areas with hard structure were located immediately adjacent to the structure on the benthic surface.

At each sampling point in the field, a 0.25m^2^ X 10 cm depth bivalve core was taken and sieved in the field through 1mm square mesh. A 10 cm diameter X 10 cm depth sediment core was taken ≤10 cm away from the bivalve core from an area with no visible debris or organisms. Both the sieved bivalve cores and the intact sediment cores were placed in separate plastic bags and frozen at -18°C until analysis.

In the lab, bivalve samples were re-sieved through 1mm square mesh using tap water to remove remaining fine sediments. Bivalves were removed using a dissecting microscope, enumerated, measured, and identified to the lowest taxonomic level possible, usually species. Bivalve community variables of total bivalve density, species richness and density of each species were calculated. Manila clam total density, density of adults (defined as ≥2 cm diameter; [6],[7],[10]) and density of juveniles (<2 cm diameter) were also calculated and used in the statistical analyses described below.

Each sediment sample was thawed and homogenized. Pre-weighed and labeled crucibles (25 ml) were filled with sediment, dried at 55°C and reweighed to calculate dry sediment weights. Samples were then combusted and weighed once cooled in a desiccator to calculate percent organic matter content. We measured the porewater salinity by filling a 10 cc syringe containing two 1 cm diameter Whatman no. 1 filter papers with a portion of surface sediment, and squeezing the porewater on to a Leica handheld salinity refractometer. Sediment texture was measured using a Beckman-Coulter LS-230 Laser Particle Sorter that calculated the percent of particles that were in <4μm, 4–63μm, and 63μm—1 mm diameter size classes (i.e., clay, silt and sand size classes). Distance, measured as direct paths of water, between each site and the mouth of the bay at the ocean was measured using Google Earth 7.1.2.2041.

Since sampling of these somewhat motile clams occurred during low tide and we wished to obtain paired samples of bivalves and environmental conditions, we estimated salinity using the porewater water of the surface sediments, which is reflective of overlying bottom water salinities [20].

Differences in the presence (or absence) of each bivalve species among sites and between the presence and absence of structure were tested using two-way ordinal logistic ANOVA. Differences in bivalve densities and sediment variables among sites and in the presence and absence of structure were determined using two-way mixed-model ANOVA using REML (restricted maximum likelihood). Block (site) was treated as a random effect, since each site was chosen haphazardly from among a large population of potential sites across the bay [25]. The mixed-model experimental design allowed us to focus on the main effects (site and presence or absence of structure), regardless of any potential interaction between these random and fixed effects [26]. Sites in which bivalve species were absent were removed from the test of differences in abundance of that particular bivalve. Relationships between Manila clam densities and environmental variables (presence or absence of hard structure, distance from mouth, and sediment variables listed in Table 1) were tested using stepwise multiple regressions with the criteria of p≤0.05 to enter the model and both p>0.05 and r^2^≤0.03 to be removed. Relationships between Manila clam density and bivalve species richness and density were tested with Pearson correlations. All analyses were run in JMP Pro 11. Data were first inspected for homogeneity of variances and normality using Kolmogorov-Smirnov and Shapiro Wilk tests, respectively; density data were 4^th^ root transformed and fraction data were arcsin square root transformed to meet assumptions of the parametric tests.

**Table 1:** Results of two-way ANOVA testing differences in sediment variables (A.) and bivalve variables (B.) between areas with and without hard structure) and among sites in Mission Bay.

Variable	Structure				Hard structure comparison		Site differences
	p	F	df	n	present	absent	
A. Sediment variables					Average (SE)		
Porewater salinity	0.33	1.0	1	60	34.3 (2)	35.4 (1)	EMB,SM≥DAC,CP≥IP,WFI,SW≥CLD,NC,NSB
%Organic matter	0.08	3.2	1	60	1.3 (0.3)	1.2 (0.7)	EMB> all others
%Sand (63μm-1mm)	0.53	0.4	1	60	80 (4)	82 (5)	SW,SM,WFI,CLD,IP,NSB,NC>DAC,EMB>CP
%Silt (4–63 μm)	0.48	0.5	1	60	18 (4)	16 (4)	CP,EMB>DAC>all others
%Clay (<4μm)	0.90	0.0	1	60	2.3 (0.5)	2.3 (0.6)	CP,EMB,DAC>all others
B. Bivalve variables					Average no. m^−2^ (SE)		
Species richness	0.41	0.7	1	32	1.7 (0.2)	2.0 (0.3)	CLD≥CP≥NSB,EMB≥SW≥DAC,NC≥SM≥IP≥WFI
Densities							
**Total bivalve**	**0.04**	**4.7**	**1**	**60**	**22.5 (6.4)**	**9.2 (3.0)**	CLD≥CP≥NSB,EMB≥SW,DAC≥NC≥SM≥IP,WFI
Total bivalve (minus *V*.*p*.)	0.56	0.4	1	60	8.3 (2.2)	7.7 (2.8)	CP≥EMB,CLD≥NSB,DAC,SW,SM≥NC≥IP,WFI
**Total *V*.*p*.**	**0.03**	**8.0**	**1**	**48**	**16.2 (5.5)**	**1.7 (0.7)**	CLD≥CP,NSB,NC,SW≥DAC,EMB,IP
**Adult *V*.*p*.**	**<0.01**	**28.0**	**1**	**36**	**14.9 (6.5)**	**0.7 (0.4)**	CP≥CLD,NSB≥DAC,NC,SW
**Juvenile *V*.*p*.**	**0.02**	**5.5**	**1**	**48**	**6.3 (2.4)**	**1.2 (0.5)**	N.S.
*Chione fluctifraga* (Veneridae)	0.47	0.5	1	42	0.8 (0.4)	1.3 (0.6)	N.S.
*Chione undatella* (Veneridae)	0.21	1.6	1	36	4.4 (1.4)	1.6 (0.6)	N.S.
*Musculista senhousia* (Mytilidae)	0.73	0.1	1	36	4.0 (2.1)	4.0 (2.9)	N.S.
*Tagelus californianus* (Cultellidae)	0.22	1.8	1	12	3.3 (2.6)	7.3 (3.6)	N.S.

Structure presence/absence was treated as a fixed variable, while site (block) was designated as a random variable. N = 60 samples (6 each per 10 sites) *V*.*p*. = Manila clam (*Venerupis philippinarum*). Average (±standard error) of raw (untransformed) variables in the presence and absence of hard structure are shown; bold indicates significant difference between the presence and absence of structure. Significance was determined using Bonferroni adjusted alpha value (initial α = 0.05).

## Results

**Fig 2:**
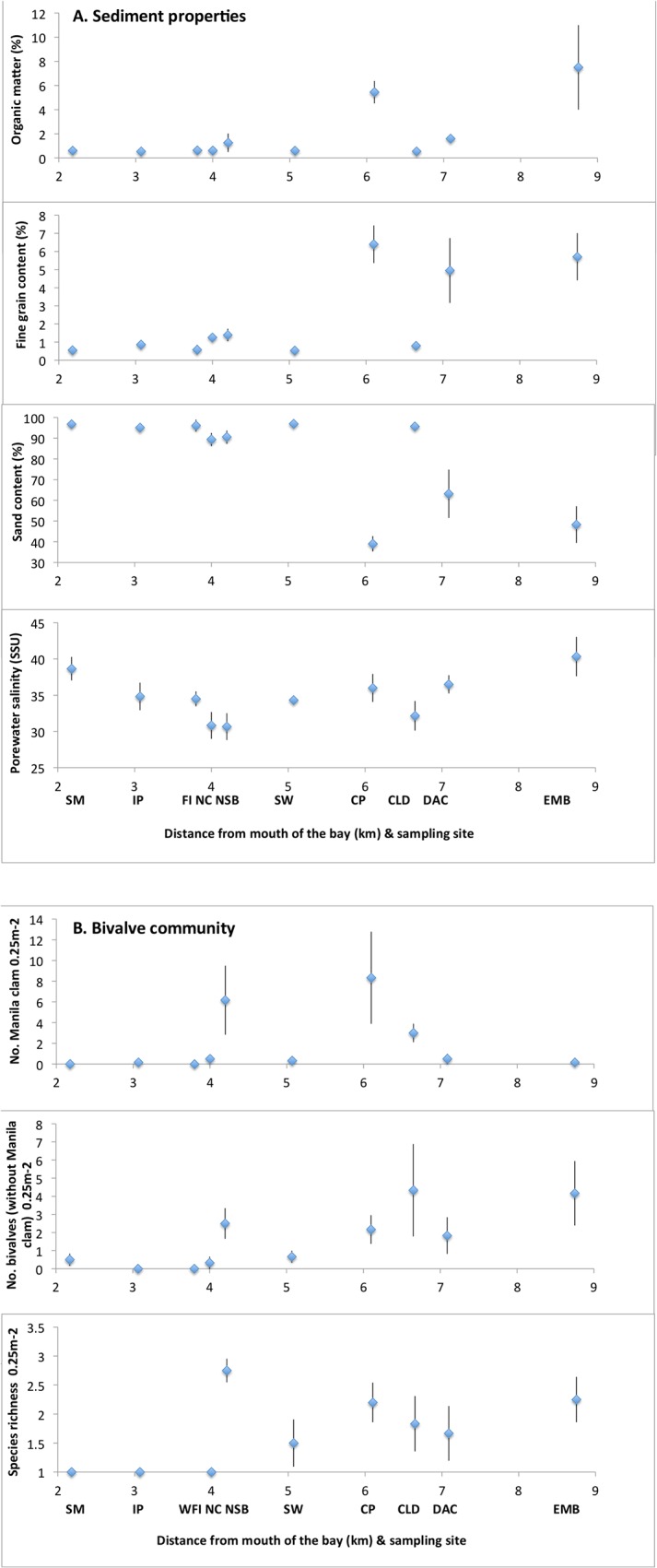
Average (±SE) sediment variables (A.) and bivalve community variables (B.) in each study site within Mission Bay, San Diego, California, USA. N = 6 samples per site. Fine grained sediment content refers to % silt + % clay.

Bay environmental conditions. Sediment texture, organic matter content and porewater salinity were similar between areas with and without hard structure, and only differed with site, but with no strong estuarine gradients from mouth to back of the bay (Table 1A, Fig. 2A). The sites with highest proportions of fine (silt and clay), organic sediments were located in areas with relatively low flushing at the back of the bay (e.g., DAC, EMB; Table 1A; Fig. 1) and/or associated with salt marsh (CP). The highest porewater salinities were also found in relatively low flow areas (DAC, EMB, CP) or at the mouth of the bay (SM), while the lowest salinities were in the mid-bay (NC, NSB, CLD; Table 1A; Fig. 1).

Bivalve community. A total of five bivalve species were found during this study. Two introduced species were found—the Japanese mussel (*Musculista senhousia*) and the Manila clam. The rest were native species from the families Veneridae, Mytilidae and Cultellidae (Table 1B). Bivalves were present in samples from all sites except WFI (Fig. 1), The presence (or absence) of both *Chione undatella* and bivalves (as a taxon) varied among sites only (ordinal logistic 2 way ANOVA: chi square ≥41, n = 60, df = 19, p≤0.001) while the presence/absence of *Chione fluctafraga*, *M*. *senhousia* and *Tagelus californianus*, did not differ with site or structure (chi square ≤30, n = 60, p≥0.09).

The highest species richness and total bivalve density was in the northern mid to back half of the bay (CP, NSB, CLD, EMB), with no differences between areas with and without structure (Table 1B). Densities of each bivalve species except Manila clam were similar between areas with and without structure, and among sites (Table 1B.).

Bay-wide influences on the Manila clam. The presence (or absence) of Manila clam adults and juveniles was dependent on site and not on the presence or absence of structure (ordinal logistic 2 way ANOVA: chi square ≥43, n = 60, p≤0.001). Densities of the adults and juveniles were also most strongly associated with site as well as weakly negatively correlated with porewater salinity (Table 2). Of the sites where the Manila clam was present, the highest total and highest adult densities were located in the northern and back halves of the bay (CLD, CP, NSB; Fig. 1); while density of juveniles did not differ with site (Table 1B). These sites were often similar to those that contained the highest densities of other bivalves (Manila clam vs. other bivalve density: r = +0.42, p = 0.001, n = 60) and species richness (Manila clam density vs. species richness: r = +0.68, p<0.001, n = 60; Table 1B; Fig. 2B).

**Table 2:** Results of multiple linear regression testing relationships between densities of all Manila clam individuals (A.), juveniles only (<2 cm diameter) (B.), and adults only (C.); and environmental variables (presence/absence of hard structure, distance from bay mouth, sediment salinity, grain size and organic matter content) in Mission Bay, San Diego, California, USA.

A. All individuals					
Predictor	R^2^/r^2^	P/p	F	df	n
Whole model	0.44	<0.001	22.1	2,57	60
Site	0.38	<0.001			
Porewater salinity	−0.06	0.02			
B. Juveniles (<2 cm diameter)					
Predictor	R2/r2	P/p	F	df	n
Whole model	0.37	<0.001	16.8	2,57	60
Site	0.33	<0.001			
Porewater salinity	−0.04	0.05			
C. Adults (≥2 cm diameter)					
Predictor	R2/r2	P/p	F	df	n
Whole model	0.46	<0.001	15.9	2,57	60
Site	0.47	<0.001			
Porewater salinity	−0.06	0.02			

**Fig 3:**
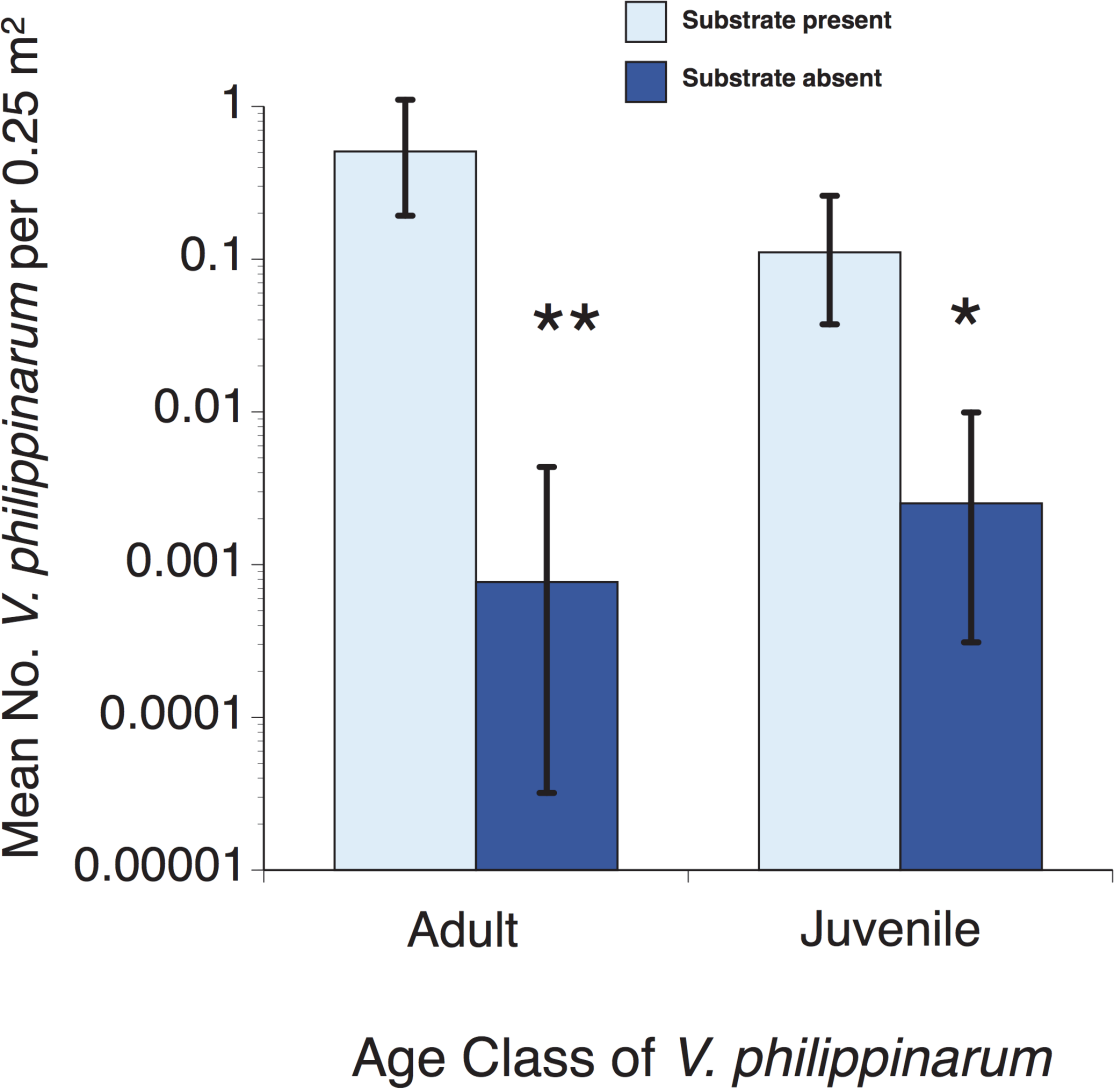
Backtransformed average (±SE) density of the Manila clam in benthic cores taken from beside hard substrate (rip rap) and in adjacent areas without significant hard substrate in Mission Bay, San Diego, California, USA. n = 10 paired sites. * = 0.01<p<0.05, ** = p<0.01.

Influences of hard structure within sites on the Manila clam. Of all the bivalves found, only densities of the Manila clam were significantly higher in association with hard structure (Fig. 3); this was true of both mature (≥2 cm diameter) and juvenile (< 2 cm) individuals (Table 1B; Fig. 3).

## Discussion

### Bay-wide distribution

Site had the strongest association with bay-wide distributions of the Manila clam. Relationships between the clam and the environmental variables we measured were lacking or weak suggesting that site variables not measured in this study, such as rates and timing of freshwater inputs, water quality, pollutants, or predation rates, may be influencing distributions of the clam [16],[27–30]. Like other marine organisms, distribution of the clam is also likely influenced by chance associated with variability in freshwater inputs and currents, and the availability of suitable substrate at the time of transport and settlement (e.g., [31], [32]). Sites that supported mid to high densities of the Manila clam also tended to have some of the highest overall bivalve densities and species richness, a pattern that was also observed in Los Angeles County [12], signifying that those sites have properties that are broadly amenable to a variety of bivalve species. For example, these sites occur along paths of circulation in the bay likely resulting in passive transport of larvae into these areas [33]. The sites may also have environmental conditions that are suitable for a diversity of bivalve species, such as relatively low physical stress and disturbance (e.g., little desiccation, salt stress or sedimentation; [34]), acceptable water quality, and/or moderate to high productivity (e.g., [35]).

Salinity is well-documented as influencing larval retention and settlement rates [17],[36], which is consistent with our finding of salinity being a correlate of juvenile and adult clam density, albeit weak. Porewater salinity in Mission Bay ranged from about 30–40 PSU, which reflects an overlap with the optimal growth range of 24–31 PSU of seawater [6],[10]. Observed salinities did not reveal the dips in salinity (13–17 PSU) associated with increased retention and settlement rates revealed in lab [17] and modeling [36] studies. However, our average summertime, shallow porewater salinities of 35 PSU (i.e., average ocean salinity) or less found in six sites indicate influence of freshwater inputs with potential salinity dips during the rainy season [21] that may aid larval retention and settlement. Areas within a passive transport range but without salinity reductions had much lower larval retention rates due to larval behavior and subsequently lower adult densities [36]. Therefore, spatially and temporally dynamic patterns of salinity associated with freshwater inflows, tidal influence, and bay currents may influence dispersal patterns, rates of retention, settlement and establishment, and ultimately distributions of the Manila clam.

While the Manila clam is often associated with coarse substrates, such as sand, gravel and shell [6],[10],[37], sediment grain size had no observed effect on distributions of the Manila clam in this study where grain sizes were ≤1 mm diameter. This was consistent with the results of lab trials revealing a lack of influence of sand grain size classes ranging from 63 μm to 2 mm diameter on settlement rates [17]. When larger materials such as gravel or shell hash are offered, however, survival rates and density are greater than on natural mudflat substrates [37]. The lack of these coarse substrates from Mission Bay and other Southern California bays, however, does not appear to limit invasion of the clam indicating that the habitat requirements which are filled by coarse substrate may also be filled by large, hard structures.

### Hard structure effects

Within tidal flat sites, the presence of hard structure had the strongest positive influence on Manila clam density in this study. It is well known that the addition of hard structure to a soft environment, such as riprap or pier pilings, can change the community through provision of substrate for fouling and open coast organisms [38],[39]. Hard structure may also alter the underlying sediment surface through loss of area, shading, refuge creation, and changes to hydrodynamic and sediment transport patterns, all of which influence the benthic community beneath and adjacent to the structure [37],[40–42].

The specific mechanism for facilitation of the Manila clam by riprap in this study is uncertain, but could include some of these explanations. Hard structure can have a number of indirect influences on species establishment. Depending on the shape and location of the structure relative to currents, structure can lead to increased turbulence, scour and coarser sediments [40], or baffling of currents and accumulation of fine sediment particles and organics [43]. Slowing of currents may also increase the likelihood of larval entrapment and/or settlement [44], and creation of “seasoned” substrate where thin layers of fine particles and organic debris that settle on coarse gravel and shell substrate increase larval settlement rates [37]. Furthermore, the cover provided by hard structure may act as predator and physical stress refuge for the shallow, sometimes mid-intertidal dwelling Manila clam. The clam is subject to heavy shorebird predation in some regions [28] and likely experiences predation by rays and crabs [6], [10], [29]. The overhangs and crevices of hard structures may also provide shade and trap moisture likely reducing desiccation risk for the clam.

While the mechanism is unclear, these results demonstrate that human alteration of the habitat, specifically the addition of hard structure, facilitates the Manila clams’ presence in Mission Bay. With sea level rise adaptation planning underway in this and many areas, the prevalence of armored shorelines may be on the rise, and existing terrestrial hard structure will increasingly come in contact with the intertidal. The addition of more hard structure on our soft shorelines has the potential to facilitate this benthic dweller, in addition to other introduced species (e.g., [45]). Alternatively, proposals of living shorelines, such as introduction of native oyster beds on tidal flats throughout this region [46], [47], may also provide coarse substrates that could facilitate spread (e.g., [6]). More information is needed on the distributions and spreading potential of the Manila clam in its southern range to make informed decisions.

In particular, we need more information on the life history and growth of the clam in its southern range, where warmer conditions may lead to faster growth and more frequent spawning [7],[48]. We also need to better understand dispersal, settlement and establishment patterns in space and time as related to freshwater pulses, other environmental variables (e.g., water quality, pollution, disturbance, parasite load; [28],[49], and behavior [34],[50]. A regional approach to data collection is needed throughout the Southern end of this introduced range to better understand how these factors change with location and conditions, and to document current distribution against which to measure spread.

While effects of the Manila clam on other species along the west coast of North America are only recently starting to emerge [51], studies from Europe reveal potential dramatic ecosystem-level effects associated with dense beds of the clam [52],[53]. Dense beds and dominance by this clam have been documented in San Francisco Bay and Colorado Lagoon in Los Angeles County (e.g., about 800–1000 m^−2^ in productive beds, [10],[12]), and several relatively dense patches have been observed in other estuaries throughout Southern California ([24], H.M. Page pers. comm.). Tracking the habitat requirements, spread and establishment of the Manila clam will inform management priorities and actions in this and other regions, which is especially important given the various plans being considered to adapt with climate change.

## References

[1] Lockwood J, Hoopes MF, Marchetti M (2007) Invasion Ecology Malden, MA: Blackwell Publishing. 312 p.

[2] Carlton J (1992) Introduced marine and estuarine mollusks of North America: an end-of-the-20^th^-century perspective. J Shellf Res 11: 489–505.

[3] Silliman BR, Bertness MD (2004) Shoreline development drives invasion of *Phragmites australis* and the loss of plant diversity on New England salt marshes. Conserv Biol 18: 1424–1434.

[4] Gedan KB, Silliman BR, Bertness MD (2009) Centuries of human-driven change in salt marsh ecosystems. Annu Rev Mar Sci 1: 117–141. 10.1146/annurev.marine.010908.163930 21141032

[5] Johnson K (2008) Culture of clams In Larinto T, editor. Status of Fisheries Report: A Report to California Fish and Game Commission as directed by the Marine Life Management Act of 1998. Sacramento: California Department of Fish and Game pp 19.1–19.5. Available: http://www.dfg.ca.gov/marine/status/. Accessed 2013 Sept 30.

[6] Cohen AN (2011) The Exotics Guide: Non-native Marine Species of the North American Pacific Coast Center for Research on Aquatic Bioinvasions and San Francisco Estuary Institute Available: http://www.exoticsguide.org. Accessed 2013 Oct 15.

[7] Bourne N (1982) Distribution, reproduction and growth of Manila clam, *Tapes philippinarum* (Adams and Reeves) in British Columbia. J Shellfish Res 2: 47–54.

[8] Chew K (1989) Manila clam biology and fishery development in western North America In Manzi JJ, Castagna M, editors. Clam mariculture in North America. Developments in Aquaculture and Fisheries Science. Amsterdam: Elsevier pp. 243–261.

[9] Jones GG, Sanford CL, Jones BL (1993) Manila clams: Hatchery and Nursery Methods Skerry Bay, B.C. Canada: Innovative Aquaculture Products Ltd Available: http://www.innovativeaqua.com. Accessed 2013 Sept 29.

[10] Reilly PN (2001) Littleneck clams In Leet WS, Dewees CM, Klingbeil R, Larson EJ, editors. California’s Living Marine Resources: A Status Report. Sacramento: The Resources Agency, The California Department of Fish and Wildlife Available: http://www.dfg.ca.gov/marine/status/status2001.asp. Accessed 2013 June 12.

[11] Tuskes PM (2012) Survey of Mission Bay mollusks, San Diego, California. The Festivus XLIV(2): 13–29.

[12] Burnaford JL, Henderson SY, Pernet B (2011) Assemblage shift following population collapse of a non-indigenous bivalve in an urban lagoon. Mar Biol 158: 1915–1927.

[13] MacDonald KB (1969) Quantitative studies of salt marsh mollusk faunas from the North American Pacific Coast. Ecol Monogr 39: 33–60.

[14] Crooks JA (2001) Assessing invader roles within changing ecosystems: historical and experimental perspectives on an exotic mussel in an urbanized lagoon. Biol Invasions 3: 23–36.

[15] Grosholz E, Crafton RE, Fontana RE, Pasari J, Williams S, et al. (2012) Aquatic Invasive Species Vector Risk Assessments: An Analysis of Aquaculture as a Vector for Introduced Marine and Estuarine Species in California. A Final Report by Bodega Marine Laboratory and Department of Environmental Science and Policy at University of California Davis submitted to the California Ocean Science Trust and Funded by the Ocean Protection Council. Available: http://calost.org/pdf/science-initiatives/ais/AIS_FINALOrnamentalReport.pdf. Accessed 2013 Dec 12.

[16] Fisheries and Aquaculture Department (2011) Database on Introductions of Aquatic Species (DIAS). Fishery Records Collections. FIGIS Data Collection. FAO Fisheries and Aquaculture Department. Available: http://www.fao.org/fishery/culturedspecies/Ruditapes_philippinarum/en. 2014 May 2.

[17] Tezuka N, Kanematsu M, Asami K, Sakiyama K, Hamaguchi M, et al (2013) Effect of salinity and substrate grain size on larval settlement of the asari clam (Manila clam, *Ruditapes philippinarum*). J Exp Mar Biol Ecol 439: 108–112.

[18] Nordby C, Zedler JB (1991) Responses of fish and macrobenthic assemblages to hydrologic disturbances in Tijuana Estuary and Los Peñasquitos Lagoon, California. Estuaries 14: 80–93.

[19] Levin LA, Talley TS (2000) Influences of vegetation and abiotic environmental factors on salt marsh invertebrates In Weinstein MP, Kreeger D, editors. Concepts and Controversies in Tidal Marsh Ecology. New York: Springer pp. 661–707.

[20] Lindberg SE, Harriss RC (1973) Mechanisms controlling pore water salinities in a salt marsh. Limnology and Oceanography 18: 788–791.

[21] Largier JL, Smith SV, Hollibaugh JT (1997) Seasonally hypersaline estuaries in Mediterranean-climate regions. Est Coast Shelf Sci 45: 789–797.

[22] Fimrite P (2012) Once-abundant West Coast oysters near extinction. SFGate, Friday 06 July 2012. Available: http://www.sfgate.com/science/article/Once-abundant-West-Coast-oysters-near-extinction-3689709.php#photo-3165594. Accessed: 2014 Jan 14.

[23] Ermgassen PSEZ, Spalding MD, Blake B, Coen LD, Dumbauld B, et al (2012) Historical ecology with real numbers: past and present extent and biomass of an imperiled estuarine habitat. Pro R Soc B 279: 3393–3400. 22696522 10.1098/rspb.2012.0313 PMC3396889

[24] Novoa A, Talley D, Talley TS (2013) Examining bivalve community shifts in several bays in San Diego, California and Northern Baja California, Mexico. Proceedings of the 2013 SACNAS National Conference. Available: http://sacnas.org/events/national-conf.

[25] Green BF, Tukey JW (1960). Complex analyses of variance: general problems. Psychometrika 25: 127–152.

[26] Steel RGD, Torrie JH, Dickey DA (1997). Principles and Procedures of Statistics McGraw-Hill, 672 pp.

[27] Bayne BL, Moore MN, Widdows J, Livingstone DR, Salkeld P, et al (1979). Measurement of the Responses of Individuals to Environmental Stress and Pollution: Studies with Bivalve Molluscs [and Discussion]. Philos T Roy Soc B 286: 563–581. 40278 10.1098/rstb.1979.0046

[28] Caldow RWG (2007) Benefits to shorebirds from invasion of a non-native shellfish. Pro R Soc B 274: 1449–1455. 10.1098/rspb.2007.0072 17412684 PMC2176204

[29] Dudas SE, McGaw IJ, Dower JF (2005) Selective crab predation on native and introduced bivalves in British Columbia. J Expt Mar Biol Ecol 325: 8–17.

[30] Paul-Pont I, de Montaudouin X, Gonzalez P, Soudant P, Baudrimont M (2010) How life history contributes to stress response in the Manila clam *Ruditapes philippinarum* . Environ Sci Pollut Res 17: 987–998. 10.1007/s11356-009-0283-5 20099041

[31] Siegel DA, Mitarai S, Costello CJ, Gaines SD, Kendall BE, et al (2008) The stochastic nature of larval connectivity among nearshore marine populations. PNAS 105: 8974–8979. 10.1073/pnas.0802544105 18577590 PMC2449349

[32] Bonsall MB, Hastings A (2004) Demographic and environmental stochasticity in predator–prey metapopulation dynamics. J Anim Ecol 73:1043–1055.

[33] Levin LA (1983) Drift tube studies of bay-ocean water exchange and implications for larval dispersal. Estuaries 6: 364–371.

[34] Menge BA, Sutherland JP (1987) Community regulation: variation in disturbance, competition, and predation in relation to environmental stress and recruitment. Am Nat 130: 730–757.

[35] Souza W (1979) Disturbance in marine intertidal boulder fields: The nonequilibrium maintenance of species diversity. Ecology 60: 1225–1229.

[36] Herbert RJH, Willis J, Jones E, Ross K, Hübner R, et al (2012) Invasion in tidal zones on complex coastlines: Modeling larvae of the non-native Manila clam, *Ruditapes philippinarum*, in the U.K. J Biogeogr 39: 585–599.

[37] Thompson DS (1995) Substrate additive studies for the development of hardshell clam habitat in the waters of Puget Sound in Washington State: An analysis of effects on recruitment, growth and survival of the Manila clam, *Tapes philippinarum*, and on the species diversity and density of existing benthic organisms. Estuaries 18: 91–107.

[38] Davis JL, Levin LA, Walther SM (2002) Artificial armored shorelines: sites for open-coast species in a southern California bay. Mar Biol 140: 1249–1262. 10.1007/s00227-002-0779-8

[39] Wetzel MA, Scholle J, Teschke K (2014) Artificial structures in sediment-dominated estuaries and their possible influences on the ecosystem. Mar Env Res 10.1016/j.marenvres.2014.04.008 24816192

[40] Davis N, VanBlaricom GR, Dayton PK (1982) Man-made structures on marine sediments: effects on adjacent benthic communities. Mar Biol 70:295–303. 10.1007/BF00396848

[41] Barros F, Underwood AJ, Lindegarth M (2001) The influence of rocky reefs on structure of benthic macrofauna in nearby soft-sediments. Est Coast Shelf Sci 52: 191–199.

[42] Dugan JE, Hubbard DM, Rodil IF, Revell DF, Schroeter S (2008) Ecological effects of coastal armoring on sandy beaches. Mar Ecol 29 (s1): 160–170. 10.1111/j.1439-0485.2008.00231.x

[43] Peterson CH, Luettich RA, Micheli F, Skilleter GA (2004) Attenuation of water flow inside seagrass canopies of differing structure. Mar Ecol Prog Series 268: 81–92. 10.3354/meps268081

[44] Abelson A, Denny M (1997) Settlement of marine organisms in flow. Ann Rev Ecol Syst 28: 317–339.

[45] Tyrrell MC, Byers JE (2007) Do artificial substrates favor nonindigenous fouling species over native species? J Exp Mar Biol Ecol 342: 54–60.

[46] Cooper M (2013) San Diego Bay Native Oyster Restoration Plan. Coastal Conservancy. Available: http://scc.ca.gov/webmaster/ftp/pdf/sccbb/2013/1306/20130620Board3H_SD_Native_Oyster.pdf. Accessed: 2014 Jan 13.

[47] Fimrite P (2013) 2 million oysters in bay begin restoration effort. SFGate, Friday 15 November 2013. Available: http://www.sfgate.com/science/article/2-million-oysters-in-bay-begin-restoration-effort-4984300.php. Accessed: 2014 Jan 14.

[48] Ponurovsky SK, Yakovlev YM (1992) The reproductive biology of the Japanese littleneck, *Tapes philippinarum* (A. Adams and Reeve 1850) (Bivalvia: Veneridae). J Shellfish Res 11: 265–277.

[49] Pellizzato MT, Galvan R, Lazzarini P, Penzo P (2011) Recruitment of *Tapes philippinarum* in the Venice Lagoon (Italy) during 2002–2007. Aquaculture International 19: 541–554. 10.1007/s10499-010-9370-3

[50] Thrush SF, Hewitt JE, Pridmore RD, Cummings VJ (1996) Adult/juvenile interactions of infaunal bivalves: contrasting outcomes in different habitats. Mar Ecol Prog Ser 132: 83–92.

[51] Bendell LI [2014] Evidence for declines in the native *Leukoma staminea* as a result of intentional introduction of the non-native *Venerupis philippinarum* in coastal British Columbia, Canada. Est Coasts 37: 369–380.

[52] Pranovi F, Franceschini G, Casale M, Zucchetta M, Torricelli P, et al (2006) An ecological imbalance induced by a non-native species: the Manila clam in the Venice Lagoon. Biol Invasions 8: 595–609.

[53] de Moura Queirós A, Hiddink JG, Johnson G, Nogueira Cabral H, Kaiser MJ (2011) Context dependence of marine ecosystem engineer invasion impacts on benthic ecosystem functioning. Biol Invasions 13: 1059–1075. 10.1093/neuonc/nor109 21813510 PMC3177664

